# On why environmental justice must continue to guide public health research

**DOI:** 10.1038/s41370-026-00865-8

**Published:** 2026-03-20

**Authors:** Jenna K. Honan

**Affiliations:** Department of Environmental Health Sciences, The University of Alabama at Birmingham, Birmingham, AL USA

“But we must recognize that the line of separation between injustice and misfortune is a political choice, not a simple rule that can be taken as a given.”—Judith N. Shklar, *The Faces of Injustice*

## We all come from somewhere

As a child, I witnessed firsthand how environmental contamination can shape a community. I grew up in the 1990s in a small mining town off the industrious Route 66 in Arizona. We were situated in the Mojave Desert near the Colorado River and Lake Mead, which were our primary sources of drinking water. Access to healthcare, higher education, healthy foods, and good paying jobs was limited. It was normal, it seemed to me at the time, to be in poor health. My family’s struggles with diabetes, obesity, cancer, and reproductive health issues mirrored those of our friends and neighbors, reflecting patterns that, even then, appeared to us to extend beyond genetics alone.

I later learned that from 1945 to 1998, the Kerr-McGee Chemical Corporation had polluted Lake Mead and the Colorado River with perchlorate, an endocrine-disrupting chemical (EDC) [1]. EDCs can mimic, block, or interfere with our natural hormones, which govern biological processes like metabolism, reproduction, and development. When we drink water contaminated with EDCs, our delicate balance of internal hormonal signals can become dysregulated [2, 3]. Even low-dose exposures over time can trigger cascading health effects, possibly contributing to adverse outcomes like obesity, diabetes, cancer, and reproductive disorders [4], the very health issues prevalent in my community [5, 6].

## The failure of reductive risk assessments

In 1997, public health officials quelled our concerns about the water pollution with seemingly straightforward reassurances. We were told by the Assistant Clark County Health Officer that, “[I]t would take a person drinking more than 250 gallons of water a day, laced with 11 parts per billion of perchlorate, to receive a significant dose affecting health… Before the perchlorate would affect the drinker, he or she would die from an overdose of water” [7]. A chief microbiologist for the Southern Nevada Water Authority stated, “Any long-term exposure to the levels of perchlorate found in Southern Nevada’s drinking water supply would be easily handled by the thyroid gland.” Nearly three decades later, we should recognize these assessments as inadequate. They focused narrowly on acute toxicity to a single organ system while ignoring potential cumulative effects, vulnerable populations (e.g., children, pregnant women), and multi-system impacts.

This reductive approach exemplifies a broader problem in public health research of the failure to account for how social, economic, and environmental factors interact to create health disparities. Environmental health scientists now strive to prevent these types of systematic biases that were once commonplace in applied research by considering how these factors affect and are affected by each distinct community. Though still not perfect, many translational researchers use methods that incorporate the unique culture and identity of each community to minimize assumptions, mitigate misinterpretation of results, and avoid inaccurate conclusions [8].

## Statistical invisibility, or the illusion of protection

Even with the understanding that a one-size-fits-all approach is insufficient, the majority of regulatory risk assessments operate under the presumption that protecting up to the 95th or even the 99th percentile represents adequate safeguarding of public health [9]. This approach, while appearing robust, fundamentally misrepresents the magnitude and distribution of harm when scaled to population-level impacts. To put this into perspective, the U.S. population is ~348 million people in 2025. If risk is considered “acceptable” when it protects 99% of people, this leaves almost 3.5 million Americans unprotected. For a 95% protection standard, this number rises to 17.5 million people, nearly the entire population of the state of New York. When agencies establish “safe” exposure levels using the 95th percentile, they implicitly acknowledge that 5% of individuals may still experience harm. Across vulnerable or sensitive subpopulations, like children under six, pregnant women, immunocompromised individuals, the elderly, migrants, LGBTQ+ individuals, those with genetic predispositions, homeless people, veterans, rural residents, people who experience physical or mental disabilities, those who are otherwise socioeconomically disadvantaged, and others [10], this percentage can be substantially higher.

Even so-called protective margins of safety often fail. Standard adjustment factors, used in risk assessments to account for insufficient data and knowledge on parameters that affect health or exposure risks, rarely reflect the full range of real-world variability [11]. For example, children drink more water, breathe more air, and eat more food per bodyweight than do adults, while their developing anatomies create windows of unique vulnerability, where even minimal exposures can cause permanent damage [12]. For EDCs like perchlorate, children are more sensitive to exposure than adults due to their exploratory behaviors (e.g., swimming, digging, and mouthing) and are more likely to experience adverse health outcomes because of their still-developing endocrine systems. Pregnant women face additional risks due to physiological changes that can increase absorption and create fetal exposure pathways. Unfortunately, the data for those who are considered “outliers” are often limited or unavailable, and the assumptions that assessors must make in their calculations, such as simply multiplying the risk estimates by 10, can lead to compounded underestimates of risk for those who are more susceptible.

The statistical models underlying risk assessments also frequently do not account for the spatial overlap of contaminants or the correlation between environmental burdens and social determinants of health. Many studies focus on a single toxicant, even though community members who experience elevated levels of one pollutant are, in reality, more often exposed to elevated concentrations of chemical mixtures via multiple pathways, more frequently, and for longer durations [13]. Industrial facilities, hazardous waste sites, and other sources of pollution cluster in communities with lower property values, reduced political capital, and limited time and resources for environmental advocacy or litigation [14]. Communities, like mine, with high environmental burdens typically experience poverty, food insecurity, inadequate healthcare access, chronic stress, and other social determinants of health that can increase exposure risk and biological sensitivity while reducing capacity to recover [15]. These oversights again lead to systematic underestimation of actual health impacts, particularly among the most vulnerable.

## One nation, indivisible

Perhaps the most fundamental limitation lies not in statistical methods but in the systematic absence of data from certain communities. Those with the highest levels of pollution tend to receive the least environmental monitoring and health surveillance [16]. In spite of this, Dr. Jay Bhattacharya, current Director of the National Institutes of Health (NIH), recently gave an interview where he indicated a priority shift away from focusing on those most susceptible to exposures or adverse health effects, instead leaning towards population-level assessments for the sake of neutrality [17]: “…[T]he NIH has as its mission the research that advances the health and longevity of all Americans, meaning everybody. It doesn’t matter what your race is, it doesn’t matter what your age is, it doesn’t matter what your sex is, it doesn’t matter what your sexual orientation is. It doesn’t matter who you are.”

In terms of effective public health measures, I contend that it matters a great deal who you are. The unprotected 1–5% does not represent a random distribution across the American population; extensive evidence demonstrates that those who fall into the unlucky tail of this distribution are systematically concentrated in specific communities, sociodemographic groups, and geographic regions. An analysis of Superfund site demographics reveals this pattern starkly. While about 41% of the U.S. population identified as an ethnic or racial minority (i.e., Hispanic and/or non-white) in 2022, minorities comprised more than 50% of people living within one mile of a Superfund site, indicating disproportionate exposure burdens on communities of color [18]. Approximately 78 million Americans live within three miles of a Superfund site, creating massive exposure cohorts that regulatory frameworks already consistently underestimate, even before this new NIH directive.

It also matters where you are. A rural farming community might have only a few hundred residents, but they will experience elevated environmental exposures if they grow crops in soil that contains mining waste, drink pesticide-contaminated groundwater from unregulated private wells, or live downwind from industrial emissions. We might also expect them to have higher-than-usual rates of respiratory illness, cardiovascular disease, or pre-term births [19, 20]. These problems would be easy to sweep under the 1–5% rug when examined at this small scale. The cumulative burden across the nation, however, becomes clear when we account for the approximately 24% of Americans (nearly one in four) living in rural areas [21], many of whom face multiple environmental stressors, have limited access to healthcare or environmental remediation resources, and tend to be older and lower-income than urban populations [22].

Our current quantification bias, especially poignant now in the Big Data era of the Information Age, still overlooks certain groups. If we are unable to recognize that public health needs are not equally divided among us, and if we are in turn unable to focus our data collection efforts in high-need communities, then we risk returning to the systematic biases and inappropriate conclusions that we have worked hard to alleviate. Not only would a community-agnostic approach potentially attenuate measurable effect sizes, normalize individual outcomes against incompatible reference points, and increase research costs by requiring the inclusion of participants less relevant to the research question, but this explicit shift toward population-representative studies also risks further marginalization of high-risk communities. Yet with Uncle Sam’s finger now placed firmly on the generalizability side of the research scale, assessments will continue to treat vulnerable populations as statistical outliers rather than priority groups requiring heightened protection efforts.

## Conclusions

Environmental health disparities exist as predictable consequences of how we conduct risk assessments, how we blanketly define acceptable risk, and how we implement environmental policy. If it’s the dose that makes the poison, then it’s the behaviors and habits that people have, and the environments in which they live, and the genetics with which they were born, and the frequency, duration, and windows of development at which they are exposed, and the continued lack of directed prevention efforts from those who consider their risks acceptable that makes the disease. Our detached ability to tolerate human loss represents a profound moral failing disguised as scientific objectivity. To co-opt the words of Oregon Senator Ron Wyden, under our current approach, we must “tell the American people how many preventable… deaths are an acceptable sacrifice for enacting an agenda that is fundamentally cruel and defies common sense” [23].

I often think about my own exposures, particularly those from my childhood, and wonder how they are connected to the health consequences I have already faced as a young woman. While the health officials from 1997 accurately predicted that my thyroid gland could “handle” the EDCs and continue to function, the rest of my endocrine system tells another story. I started menstruating at just 9 years old, and in my early 30s, I needed a hysterectomy. Because of this, I will never bear children. Instead, will I spend my time negotiating with my health insurance over why I needed to have my first diagnostic mammogram at age 35, half a decade earlier than the generally recommended 40, due to my now-elevated risk of developing breast cancer [24]? Am I, like many others from my hometown, merely waiting my turn for a diabetes diagnosis? Am I simply the “acceptable” price we pay for the sake of feasibility?

## References

[1] NDEP. Environmental Clean Up: Black Mountain Industrial (BMI) Complex. Nevada Department of Environmental Protection. 2020. https://ndep.nv.gov/environmental-cleanup/black-mountain-industrial-bmi-complex/perchlorate.

[2] Nirenjen S, Singh SA, Begum RF, Arun E, Vellapandian C, Narayanan J Exploring the impact of endocrine disruptors on hormonal regulation and adipose tissue in health and obesity. Journal of Endocrinology. 2025;266. 10.1530/JOE-24-0374. 10.1530/JOE-24-0374 40632578

[3] Parent A-S, Damdimopoulou P, Johansson HK, Bouftas N, Draskau MK, Franssen D, et al. Endocrine-disrupting chemicals and female reproductive health: a growing concern. Nat Rev Endocrinol. 2025;21:593–607. 10.1038/s41574-025-01131-x. 40404936 10.1038/s41574-025-01131-x

[4] Gupta D, Tyagi S, Singh R. Endocrine disrupting chemicals: an overview of their toxicity and implications. J Chem Health Risks. 2025;15:9–41. 10.60829/JCHR.2024.22635.

[5] Advameg Inc. Health and Nutrition of Kingman, AZ Residents. 2025. https://www.city-data.com/health-nutrition/Kingman-Arizona.html.

[6] KRMC & MCDPH. Community Health Needs Assessment. Kingman Regional Medical Center (KRMC) and Mohave County Department of Public Health (MCDPH). 2019. [https://www.azkrmc.com/sites/default/files/2019-07/PinnaclePrevention_MohaveCounty_Report_Rev_8-22-19.pdf.

[7] Las Vegas Sun. Kerr-McGree [sic] well highly contaminated Henderson, NV. 1997. https://lasvegassun.com/news/1997/sep/05/kerr-mcgree-well-highly-contaminated/.

[8] O’Mara-Eves A, Brunton G, Oliver S, Kavanagh J, Jamal F, Thomas J. The effectiveness of community engagement in public health interventions for disadvantaged groups: a meta-analysis. BioMed Central Public Health. 2015;15. 10.1186/s12889-015-1352-y. 10.1186/s12889-015-1352-y PMC4374501 25885588

[9] U.S. EPA. Data Quality Assessment: Statistical Methods for Practitioners. U.S. Environmental Protection Agency. Washington, DC: Office of Environmental Information. 2006. https://www.epa.gov/sites/default/files/2015-08/documents/g9s-final.pdf.

[10] Shi L, Stevens GD. Vulnerable Populations in the United States. 3rd ed. Hoboken, NJ: John Wiley & Sons; 2021.

[11] U.S. EPA. Child-Specific Exposure Factors Handbook. U.S. Environmental Protection Agency. Washington, DC: National Center for Environmental Assessment. 2008. https://ordspub.epa.gov/ords/eims/eimscomm.getfile?p_download_id=484738.

[12] Sly PD, Flack F. Susceptibility of children to environmental pollutants. Ann N Y Acad Sci. 2008;1140:163–83. 10.1196/annals.1454.017. 18991915 10.1196/annals.1454.017

[13] Sprinkle RH, Payne-Sturges DC Mixture toxicity, cumulative risk, and environmental justice in United States federal policy, 1980–2016. Environ Health. 2021;20. 10.1186/s12940-021-00764-5. 10.1186/s12940-021-00764-5 PMC8449500 34535123

[14] Morello-Frosch RA. Discrimination and the political economy of environmental inequality. Environ Plan C Gov Policy. 2002;20:477–96. 10.1068/c03r.

[15] Aidoo EM. Social determinants of health: examining poverty, housing, and education in widening US healthcare access disparities. World J Adv Res Rev. 2023;20:1370–89. 10.30574/wjarr.2023.20.1.2018.

[16] Brulle RJ, Pellow DN. Environmental justice: human health and environmental inequalities. Annu Rev Public Health. 2006;27:103–24. 10.1146/annurev.publhealth.27.021405.102124. 16533111 10.1146/annurev.publhealth.27.021405.102124

[17] Röhn T. Trump’s NIH Chief Lets Loose on Fauci, Vaccines and Covid Cover-Ups. Politico. 2025. https://www.politico.com/news/magazine/2025/05/14/jay-bhattacharya-nih-chief-vaccines-covid-interview-00345488.

[18] U.S. EPA. Population Surrounding 1,881 Superfund Sites. U.S. Environmental Protection Agency. Office of Land and Emergency Management. 2023. https://www.epa.gov/system/files/documents/2023-08/FY22%20Population%20Estimates%20Superfund%20Final.pdf.

[19] Johnston J, Cushing L. Chemical exposures, health, and environmental justice in communities living on the fenceline of industry. Curr Environ Health Rep. 2020;7:48–57. 10.1007/s40572-020-00263-8. 31970715 10.1007/s40572-020-00263-8 PMC7035204

[20] O’ Brien C, Kingston L, Plant BJ, Coffey A. Lung health in farming: a scoping review. J Agromedicine. 2023;28:335–45. 10.1080/1059924X.2023.2178573. 36773027 10.1080/1059924X.2023.2178573

[21] Ramakrishnan S, Suandi M. Who Lives in Rural America? U.S. Federal Housing Finance Agency, Division of Research and Statistics. 2024. https://www.fhfa.gov/blog/insights/who-lives-in-rural-america.

[22] Hendryx M, Fedorko E, Halverson J. Pollution sources and mortality rates across rural-urban areas in the United States. J Rural Health. 2010;26:383–91. 10.1111/j.1748-0361.2010.00305.x. 21029174 10.1111/j.1748-0361.2010.00305.x

[23] Hagstrom A. RFK Jr and top Dem clash during heated Senate hearing: ‘This is about kids’. FOX News Network. 2025. https://www.foxnews.com/politics/rfk-jr-top-dem-clash-during-heated-senate-hearing-this-about-kids.

[24] Brinton LA, Schairer C, Hoover RN, Fraumeni JF. Menstrual factors and risk of breast cancer. Cancer Investig. 1988;6:245–54. 10.3109/07357908809080645. 3167610 10.3109/07357908809080645

